# Springtime Peaks and Christmas Troughs: A National Longitudinal Population-Based Study into Suicide Incidence Time Trends in the Netherlands

**DOI:** 10.3389/fpsyt.2018.00045

**Published:** 2018-02-26

**Authors:** Emma Hofstra, Iman Elfeddali, Marjan Bakker, Jacobus J. de Jong, Chijs van Nieuwenhuizen, Christina M. van der Feltz-Cornelis

**Affiliations:** ^1^Academic Department of Specialised Mental Health Care, GGz Breburg, Tilburg, Netherlands; ^2^Tranzo—Scientific Center for Care and Welfare, Tilburg University, Tilburg, Netherlands; ^3^Department of Methodology and Statistics, Tilburg University, Tilburg, Netherlands; ^4^Institute for Mental Health Care, GGzE, Eindhoven, Netherlands

**Keywords:** suicide, time trends, seasonality, Christmas, Netherlands, gender, age, province

## Abstract

**Background:**

Time trends are one of the most studied phenomena in suicide research; however, evidence for time trends in the Dutch population remains understudied. Insight into time trends can contribute to the development of effective suicide prevention strategies.

**Methods:**

Time trends in national daily and monthly data of 33,224 suicide events that occurred in the Netherlands from 1995 to 2015 were examined, as well as the influence of age, gender, and province, in a longitudinal population-based design with Poisson regression analyses and Bayesian change point analyses.

**Results:**

Suicide incidence among Dutch residents increased from 2007 until 2015 by 38%. Suicide rates peak in spring, up to 8% higher than in summer (*p* < 0.001). Suicide incidence was 42% lower at Christmas, compared to the December-average (IRR = 0.580, *p* < 0.001). After Christmas, a substantial increase occurred on January 1, which remained high during the first weeks of the new year. Suicide occurred more than twice as often in men than in women. For both genders, the results indicated a spring time peak in suicide incidence and a trough at Christmas. Suicide rates were highest in the elderly (age group, 80+), and no evidence was found of a differential effect by season in the age groups with regard to suicide incidence. No interaction effect was found with regard to province of residence for both season and Christmas, indicating that no evidence was found that these time trends had differential effects in the Dutch provinces in terms of suicide incidence.

**Conclusion:**

Evidence was found for time trends in suicide incidence in the Netherlands. It is recommended to plan (mental) health care services to be available especially at high-risk moments, at spring time, and in the beginning of January. Further research is needed to explore the protective effect of Christmas in suicide incidence.

## Introduction

Worldwide, annual suicide rates are rising. Consequently national and international suicide prevention strategies have been developed, and there is also ample scientific attention to this issue (1, 2). In 2015, almost 800,000 people lost their lives due to suicide, which is equivalent to a suicide rate of 10.7 per 100,000 people (3). Suicide is now one of the leading causes of death, especially among young people between the ages of 15 and 29 years (2, 4–6). In the Netherlands, the annual suicide number has increased since 2007 from 1,353 to 1,871 in 2015: an increase of 38% (7). The tragedy of suicide concerns not only the premature loss of a human life but also its impact on family members, friends, bystanders, railway professionals, and communities (2, 6, 8, 9). Due to increasing suicide rates and the large impact on the individual and society, it is important to gain more knowledge on predictive factors of suicide. Several risk factors of suicide have already been identified. With regard to psychopathology, the risk of suicide is almost 50 times higher in in-patients—and particularly in persons with personality and affective disorders—than in the general population (10). Furthermore, time trends in suicide incidence have gained broad international consideration (11, 12). Still, evidence remains understudied for the Dutch population, and a better understanding of predictors of suicide is needed for developing more appropriate suicide prevention strategies. Insight into high-risk time frames in suicide might contribute to the refining of these strategies, such as setting up help lines and health care services available at the right moments. In this study, we examined time trends in suicide incidence in the Netherlands from 1995 to 2015. To this end, we will first give an overview of what is already known and we will end by giving the objectives of this study.

Evidence for daily and weekly patterns in suicide incidence were found (13–19). Regarding seasonal patterns in suicide incidence, Durkheim already suggested in the early 19th century that suicide incidence shows seasonal variation (20), and seasonality is now one of the most studied phenomenon in suicide research (18, 21–28). However, studies show conflicting evidence of seasonal patterns (15, 16, 29, 30). Several studies indicated one single peak in spring or (early) summer (21–23, 25–27), while others described two peaks: one in spring and another one in autumn (16, 18). Ajdacic-Gross and colleagues assessed the long-term change of seasonality in suicide and found that seasonality is about to fade away (31). With regards to the Netherlands, one would expect to find seasonality in suicide incidence, as the Netherlands is located at a latitude of 52°23′ N and therefore has pronounced seasons. However, two studies about seasonal patterns in suicide in Dutch residents showed significant seasonal variation in suicide incidence, with a peak in spring (32, 33), but this could not be replicated in a more recent study on train suicides (13). Therefore, the literature on seasonal patterns in suicide rates in the Netherlands is ambiguous in this respect.

As for winter-holiday trends in suicide, legend has it that suicide rates are increased at Christmas. The Annenberg Public Policy Center of the University of Pennsylvania found that many news stories in the past 17 years supported this belief (34). People may indeed experience a worsened general mood during the Christmas days because of family strains, loneliness, or seasonal affective disorder, and increased alcohol use might aggravate this (11, 35–37). On the other hand, two literature reviews reported lower suicide rates at Christmas (11, 38), and this was also reported in multiple retrospective database studies (18, 19, 39–43). A remarkable finding in these studies is that suicide rates seem to be higher on New Year’s Day and January 2, suggesting that suicides may be delayed until after the Christmas-holidays (18, 19, 40–43). Christmas is a Christian holiday, and in countries with other predominant religions, one would expect other trends. Indeed, in Turkey—where 99% of the population is Muslim—fewer suicides were observed in the Islamic holy month of Ramadan (44). However, Christians have become a minority of about one-third of the Dutch population due to secularization and the increase of immigrant religions in the Netherlands (45). The Christmas festivities might still play a protective role since Christmas is also a popular holiday in the non-religious population. The association between Christmas and suicide incidence in the Netherlands therefore yet remains unclear.

Seasonal trends in suicide and the association with gender report either no association (21, 23, 25) or a stronger association in men (16, 22, 27, 46). Some studies indicate one single peak in spring/summer for men and one peak in spring and a second minor peak in autumn for women (24, 29, 46). This “bimodal distribution” in suicide among women is, however, not found by others (22, 26, 47). Findings regarding age and seasonality are ambiguous as well, finding no association (23) or a stronger association in younger age groups (16, 25) or in older age groups (24). Evidence also suggests that the seasonal effect on suicide incidence differs across specific regions, as it is has been found that seasonality is more pronounced in rural settings than in urban settings (20, 25, 46). Furthermore, especially rural Catholic regions show seasonal patterns in suicide incidence as urban Protestant regions showed a larger decline in suicide seasonality over the last years (31). As far as the researchers know, seasonality in suicide across the provinces in the Netherlands has not yet been studied. Concerning Christmas trends, gender differences remain unclear. One study found that only men showed significant fewer suicides on Christmas day (19), while others reported a greater reduction in women (40, 43). Obviously, such data may depend on the degree to which the region where the study is performed holds religions that have Christmas celebrations. In the Netherlands, the Western and Northern areas of the Netherlands are most secularized and the percentage of immigrants is greater in the area “the Randstad,” so there is more religious plurality in this area (45). Therefore, provinces in the Netherlands may differ in terms of religion-related popularity of Christmas.

In conclusion, it remains unclear whether season and Christmas are associated with suicide incidence in the Netherlands, and if so, whether these associations differ for gender, age group, and province of residence. More knowledge of time trends is essential to refine suicide prevention strategies. These strategies are highly needed as the annual suicide rate in the Netherlands keeps rising. Therefore, this study examines time trends in suicide incidence in the Netherlands over the time period of 1995–2015. This study has four objectives:

1. To examine annual suicide incidence over the study period.
2. To examine seasonal trends in suicide incidence.
3. To examine if suicide incidence at Christmas differs from other days in December.
4. To explore if any associations differ in relation to gender, age, and province of residence.

## Materials and Methods

### Study Design

This is a retrospective longitudinal population-based study on the national register of natural and unnatural deaths data, as registered by Statistics Netherlands, a Dutch governmental institution. Statistics Netherlands receives all death certificates about injury deaths from the police and coroners, i.e., medical doctors who work in the municipal health service. In the Netherlands, a death certificate after a suicide is certified based on strict guidelines involving several professionals (48, 49). Daily and monthly data about all suicide events between 1995 and 2015, as well as age, gender, and province, were included.

### Variables

This study included four variables: (1) the occurrence of a completed suicide, and the persons’ (2) gender, (3) age group, and (4) province of residence. In this study, a completed suicide was defined as an act performed by the person him/herself with the specific purpose of taking his/her own life that indeed led to death (48). Thus, euthanasia and non-fatal suicide attempts were not included. Suicides are assigned in the StatLine registrar according to the International Statistical Classification of Diseases and Related Health Problems (ICD) of the World Health Organization as “intentional self-harm” (codes X60–X84). The 10th revision of the ICD is currently being used (48–50). Three characteristics of people who have died by suicide, and are related to suicide incidence, were also included as variables: gender, age group at time of death, and province of residence at time of death.

### Data Sources/Measurements

The data comprise 33,224 suicide events over 21 years in a population of 15.5 million in 1995 and 16.9 million in 2015. All variables were obtained from Statistics Netherlands in an Excel database and were transposed by the researchers to Statistical Package for the Social Sciences (SPSS) and R. Daily and monthly data about completed suicides that occurred in the time period of 1995 to 2015 were operationalized into seven variables: (1) total numbers of suicide for each month of each year, (2) mean number of suicide per day for each month of each year, (3) suicide rates per 100,000 residents for each month of each year, (4) daily suicide numbers by season, (5) total suicide number per day for all 21 years together, (6) daily suicide numbers at Christmas, and (7) daily suicide numbers at all other days in December (excluding Christmas). To form the total number of suicide variable, a total number of suicides was calculated for each month in each year (for example, January 1995). To form the mean number of suicides per day variable, the total number of completed suicides for each month per year was divided by the number of days in that specific month (i.e., 31 for January, March, May, July, August, October and December; 30 for April, June, September and November; and 28 or 29 for February and 29 in leap years; which were 1996, 2000, 2004, 2008, and 2012). To form the suicide rate variable, the total number of suicides was divided by that years’ mean population,^1^ which was then multiplied by 100,000. A season variable was created on all daily data according to the meteorological calendar of the Northern Hemisphere in which winter includes December, January, and February; spring includes March, April, and May; summer includes June, July, and August; and autumn includes September, October, and November. A daily total number for all 21 years taken together was created by summing all daily totals per year (for example, all January 1 of all years together).

In the Netherlands, Christmas is celebrated on December 25 and 26. Therefore, to form the Christmas variable, the daily data on December 25 and 26 were allocated to the Christmas variable and daily data on all other days in December to the non-Christmas variable. This minimizes potential seasonal variation bias when the rest of the year would be used as a control period (42). In addition to data about completed suicides, data about gender, age group, and province of residence were received. Gender was classified by Statistics Netherlands into two variables: male and female. Statistics Netherlands classified age at the time of death in the following age groups: 0–19, 20–29, 30–39, 40–49, 50–59, 60–69, 70–79, and 80+. Statistics Netherlands classified province of residence into the 12 provinces of the Netherlands.

### Bias

Since suicide is a sensitive issue, a challenge might be to obtain true incidence statistics due to the possibility of underreporting (2, 49, 51–54). The efforts that have been made to minimize this potential bias are strict methodological death registration procedures that are carried out by coroners and government institutions in the Netherlands after injury deaths. This increases the classification of suicides and decreases classification of undetermined deaths, which are the most common alternative verdicts in case of a probable suicide (49, 54). Indeed, the Netherlands belongs to the European countries with the lowest ratio of suicide to “undetermined death” (49). Hence, this possible bias is deemed to be low in this study.

### Ethics

The anonymity of the people who died by suicide was of great importance in this study. We only received and examined anonymous data of Statistics Netherlands. Consequently, we only analyzed monthly data for the age groups, as daily suicide incidences were too low within age groups. As a result, we were not able to analyze Christmas trends for age groups.

### Statistical Methods

Data preparation and statistical analyses were performed in SPSS and R. The Statement of the Strengthening the Reporting of Observational Studies in Epidemiology (STROBE) was used to transparently and completely report about our observational research study (55). Poisson regression analysis, Bayesian change point analysis, Mann–Whitney *U* test, and Kruskal–Wallis test were used to explore time trends in suicide incidence. Independent variables were day, month, year, season, Christmas days, and non-Christmas days. Dependent variables were the number of completed suicides. Demographics examined were gender, age group, and province. Gender and province were examined for yearly, seasonal, and Christmas trends, and age group was only examined for yearly and seasonal trends, as discussed in the previous paragraph. The level of significance was adjusted by Bonferroni correction. In this correction, the alpha of 0.05 was divided by the number of tests (five tests to main effects and five tests to interaction effects), resulting in an alpha of 0.005.

## Results

### National Suicide Incidence over the Period 1995 to 2015

**Figure 1:**
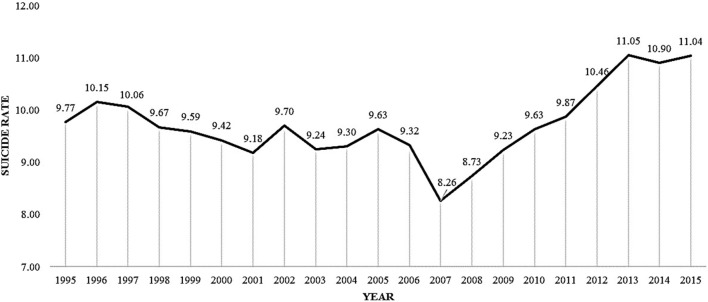
Mean suicide rates per 100,000 residents per year.

In the time period of 1995 to 2015, a total number of 33,224 residents of the Netherlands lost their lives due to suicide. The relative mean annual suicide rate in the Netherlands for all years together is 9.72 per 100,000 residents. The year with the lowest suicide rate was 2007 (8.26), and the year with the highest suicide rate was 2013 (11.05), which represents an increase of 37%. The increase in absolute suicide numbers from 2007 till 2015 was 38%, as can be seen in Figure 1.

### Seasonal Trends

A Poisson Regression Analysis on the 21-year study period taken together indicated an effect by season on suicide incidence in the population in general (χ^2^(3) = 33.250, *p* < 0.001). The mean number of suicides per day (M = 4.57, SD = 2.20) was 7–8% higher in spring than in summer (M = 4.25, SD = 2.10), autumn (M = 4.26, SD = 2.20), and winter (M = 4.25, SD = 2.16), as is shown in Table 1.

**Table 1:** Poisson regression analysis outcomes on seasonal trends in suicide.

	χ^2^	IRR	95% CI for IRR		*p*
			Lower	Upper	
Spring-Summer	22.636	1.076	1.044	1.109	0.000
Spring-Autumn	21.101	1.073	1.041	1.106	0.000
Spring-Winter	21.365	1.074	1.042	1.107	0.000
Summer-Autumn	0.023	0.998	0.968	1.029	0.880
Summer-Winter	0.012	0.998	0.968	1.029	0.911
Autumn-Winter	0.001	1.001	0.970	1.032	0.969

*IRR, incidence rate ratio*.

### Christmas Trends

**Figure 2:**
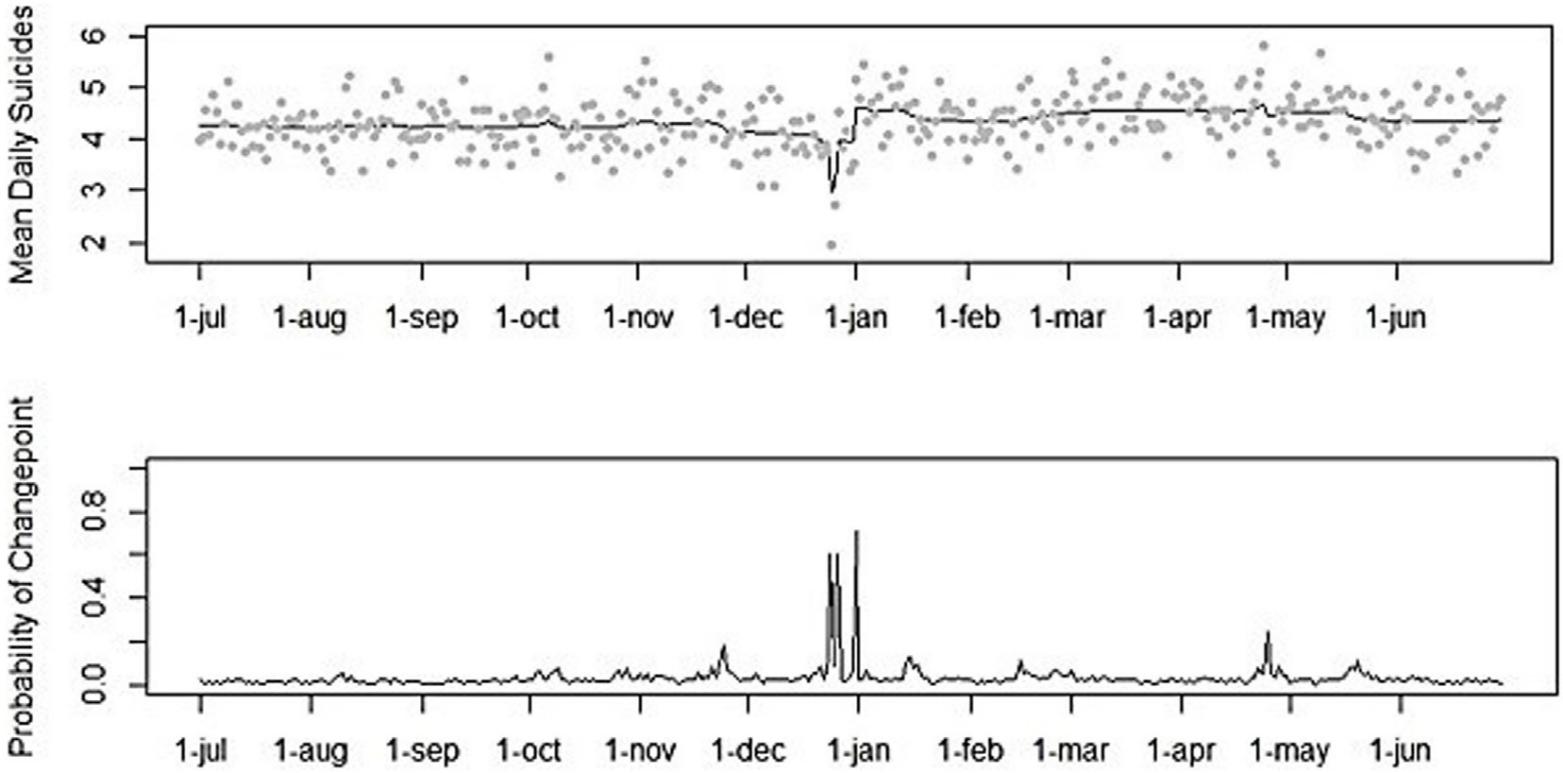
Mean numbers of suicide per day and posterior probabilities of Bayesian change point analysis (1995–2015).

**Figure 3:**
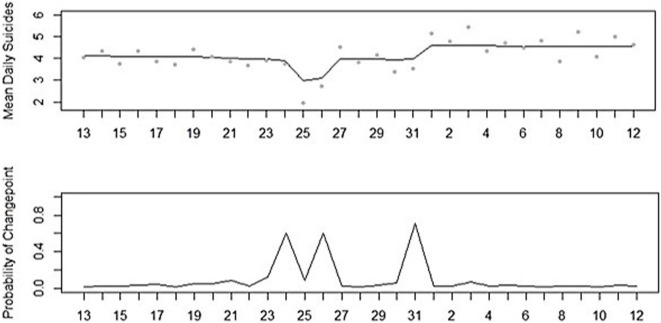
Mean numbers of suicide per day and posterior probabilities of Bayesian change point analysis for December 13 to January 12 (1995–2015).

An effect by Christmas was found in the population in general, as indicated by Poisson regression analysis on the total study period taken together (χ^2^(1) = 27.876, IRR = 0.580, 95% CI = 0.474–0.710, *p* < 0.001). Suicide incidence was 42% lower at Christmas (M = 2.33, SD = 1.39), in comparison to other days in December (M = 4.02, SD = 2.02). This effect was also indicated by Bayesian change point analysis on the daily distribution of suicide for the 21-year study period taken together. Bayesian change point analysis identified three large peaks in the posterior probabilities (PPs) of change points. First, a peak in the PP of change points on December 25 was found (*PP* = 0.608), indicating a substantive decrease in mean daily suicides. Second, a peak in the PP of change points on December 27 was found (*PP* = 0.600), indicating a substantive increase to the average daily suicide incidence in December. Third, a peak in the PP change points on January 1 was found (*PP* = 0.712), indicating a substantive increase that remained high during the first weeks of the new year, as can be seen in Figures 2 and 3.

### Associations with Age, Gender, and Province

Associations of suicide incidence with gender, age group, and province were examined. The results are provided in Tables 2–4 and discussed per demographic variable.

**Table 2:** Suicide incidence and significance levels by gender, age group, and province.

	Total numbers		Mean rates^a^	Test^b^	
	*N*	%	M	Value	*p*
**Gender**				0.000	0.000
Males	22,609	68.05	13.37		
Females	10,615	31.95	6.16		
**Age group**				133.295	0.000
0–19	981	2.95	1.20		
20–29	3,677	11.07	8.32		
30–39	5,532	16.65	11.00		
40–49	7,502	22.58	14.47		
50–59	6,830	20.56	14.98		
60–69	4,181	12.58	12.24		
70–79	2,655	7.99	11.85		
80+	1,866	5.62	15.37		
**Province**				89.730	0.000
Groningen	1,388	4.18	11.55		
Friesland	1,391	4.19	10.40		
Drenthe	1,051	3.16	10.42		
Overijssel	2,157	6.49	9.29		
Flevoland	551	1.66	7.36		
Gelderland	3,905	11.75	9.46		
Utrecht	2,179	6.56	8.88		
Noord-Holland	5,708	17.18	10.44		
Zuid-Holland	6,410	19.29	8.82		
Zeeland	797	2.40	10.06		
Noord-Brabant	5,206	15.67	10.30		
Limburg	2,481	7.47	10.45		

*^a^Per 100,000 residents*.

*^b^Mann–Whitney U test and Kruskal–Wallis test were executed on the rates*.

**Table 3:** Mean numbers of suicide per day by season for gender, age group, and province.

	Winter		Spring		Summer		Autumn		Poisson	
	M	SD	M	SD	M	SD	M	SD	χ^2^	*p*
**Gender**										
Males	2.90	1.78	3.10	1.77	2.93	1.75	2.86	1.76	21.098	0.000
Females	1.36	1.15	1.47	1.24	1.31	1.14	1.39	1.19	18.675	0.000
**Age group^a^**										
0–19	0.13	–	0.13	–	0.11	–	0.13	–	3.927	0.269
20–29	0.49	–	0.50	–	0.46	–	0.46	–	4.383	0.223
30–39	0.74	–	0.75	–	0.68	–	0.71	–	6.262	0.100
40–49	0.92	–	1.01	–	1.00	–	0.98	–	12.432	0.006
50–59	0.89	–	0.94	–	0.87	–	0.86	–	9.719	0.021
60–69	0.52	–	0.59	–	0.54	–	0.54	–	11.401	0.010
70–79	0.33	–	0.40	–	0.33	–	0.33	–	22.466	0.000
80+	0.23	–	0.25	–	0.25	–	0.24	–	3.695	0.296
**Province**										
Groningen	0.19	0.45	0.19	0.44	0.17	0.42	0.17	0.41	3.791	0.285
Friesland	0.18	0.41	0.19	0.44	0.19	0.43	0.17	0.42	1.596	0.660
Drenthe	0.13	0.37	0.15	0.39	0.13	0.37	0.13	0.37	5.270	0.153
Overijssel	0.27	0.53	0.30	0.54	0.27	0.52	0.28	0.52	2.857	0.414
Flevoland	0.07	0.27	0.07	0.27	0.07	0.26	0.08	0.28	2.872	0.412
Gelderland	0.52	0.71	0.53	0.71	0.51	0.71	0.49	0.70	3.351	0.341
Utrecht	0.27	0.53	0.30	0.55	0.27	0.51	0.30	0.54	6.500	0.090
Noord-Holland	0.72	0.88	0.78	0.90	0.75	0.88	0.73	0.87	5.040	0.169
Zuid-Holland	0.81	0.90	0.88	0.95	0.81	0.90	0.84	0.95	7.774	0.051
Zeeland	0.11	0.34	0.12	0.35	0.10	0.33	0.09	0.30	10.225	0.017
Noord-Brabant	0.67	0.82	0.72	0.84	0.66	0.81	0.66	0.84	6.393	0.094
Limburg	0.32	0.59	0.34	0.58	0.31	0.55	0.32	0.59	2.758	0.430

*^a^No SD is provided since we only had monthly data for age groups*.

**Table 4:** Mean numbers of suicide per day by Christmas for gender and province.

	Christmas days		Non-Christmas days		Poisson	
	M	SD	M	SD	χ^2^	*p*
**Gender**						
Males	1.55	1.17	2.73	1.73	20.084	0.000
Females	0.79	0.90	1.29	1.08	7.841	0.005
**Province**						
Groningen	0.05	0.22	0.19	0.44	3.635	0.057
Friesland	0.19	0.40	0.15	0.37	0.360	0.549
Drenthe	0.10	0.30	0.14	0.38	0.524	0.469
Overijssel	0.10	0.30	0.23	0.47	2.921	0.087
Flevoland	0.02	0.15	0.06	0.26	0.904	0.342
Gelderland	0.40	0.77	0.54	0.69	1.319	0.251
Utrecht	0.07	0.26	0.27	0.52	5.237	0.022
Noord-Holland	0.36	0.53	0.65	0.86	5.145	0.023
Zuid-Holland	0.43	0.63	0.73	0.83	4.841	0.028
Zeeland	0.14	0.35	0.09	0.30	1.052	0.305
Noord-Brabant	0.26	0.45	0.68	0.81	9.849	0.002
Limburg	0.21	0.47	0.29	0.59	0.856	0.355

#### Gender

First, over the 21 years together, men had significant higher mean suicide rates (M = 13.37) than women (M = 6.16) (*U* = 0.000, *p* < 0.001), as can be seen in Table 2. The subgroup effects of season on suicide incidence by gender are presented in Table 3. The interaction term, season by gender, was non-significant (χ^2^(3) = 6.634, *p* = 0.085), indicating that no evidence for a differential effect of season in the two genders with regard to suicide incidence was found. In Table 4, the subgroup effects of Christmas on suicide incidence by gender are given. The interaction term, Christmas by gender, was non-significant (χ^2^(1) = 0.100, *p* = 0.751), suggesting that–with regard to suicide incidence—there is no evidence for a differential effect of Christmas in men and women.

#### Age

As shown in Table 2, a significant age difference in mean suicide rates was found (χ^2^(7) = 133.295, *p* < 0.001). People in the age group 0–19 years (M = 1.20) and people in the age group 20–29 years (M = 8.32) had the lowest mean suicide rates. The age groups with the second highest mean suicide rates were 30–39 years (M = 11.00), 60–69 years (M = 12.24), and 70–79 years (M = 11.85). People in the age groups 40–49 years (M = 14.47), 50–59 years (M = 14.98), and older than 80 years (M = 15.37) had the highest mean suicide rates. Subgroup effects of season on suicide incidence by age groups are shown in Table 3. No interaction effect between season and age group was found (χ^2^(21) = 29.883, *p* = 0.094), indicating that no evidence was found that season had a differential effect in the age groups with regard to suicide incidence.

#### Province

As can be seen in Table 2, a significant difference in mean suicide rates across provinces was found (χ^2^(11) = 89.730, *p* < 0.001). Groningen was the province with the highest suicide rates (M = 11.55) and Flevoland was the province with the lowest suicide rates (M = 7.36). In Table 3, the subgroup effects of season on suicide incidence by provinces are presented. No interaction effect was found with regard to season and province (χ^2^(33) = 25.391, *p* = 0.825), suggesting that no evidence was found that season had a differential effect in the provinces in terms of suicide incidence. Subgroup effects of Christmas by province are given in Table 4. The interaction term, Christmas by province, was non-significant (χ^2^(11) = 16.253, *p* = 0.132), indicating that no evidence was found for a differential effect by Christmas in the provinces with regard to suicide incidence.

## Discussion

### Key Findings

In this study, time trends in suicide incidence in the Netherlands in 1995–2015 were examined. The first objective is to examine national suicide incidence over the total study period. The results indicated that suicide incidence among Dutch residents increased since 2007. These findings are in line with existing literature (2, 7). As suicide is associated with lower socioeconomic status, unemployment, evictions, and indebtedness, the increase in suicide rates is possibly associated with the global economic crisis, which started in 2007 (56–58). Psychiatric disorders—and in particular major depression—increases the risk of suicide and might, also during economic crisis, mediate the relationship between suicide and economic situation (56, 59). However, a relationship between economic crisis, psychiatric disorders, and suicide remains partly unexplained due to conflicting evidence. The loss of a job might, for example, lead to depression, which might lead to suicide, but it is also possible that people with certain mental illnesses are more prone for suicide and also for losing their job (56). Moreover, some studies fail to find an association between suicide rates and the economic crisis (56).

The second objective is to examine seasonal trends in suicide incidence. The results indicated seasonal trends in suicide incidence in the Netherlands with a peak in spring. Therefore, we conclude that seasonality in suicide has not (yet) faded away in the Netherlands. Compared to summer, autumn, and winter, in spring, there is one suicide almost every 3 days. This spring-peak is also found in studies in other countries (18, 21–27, 33). However, the study of van Houwelingen to seasonal trends in train suicides did not find any seasonal effects in the Dutch population (13). This may indicate that train suicides do not follow a seasonal pattern, but suicides in general do.

Two classical theories about the seasonal patterns in suicide exist. First, Durkheim had a sociological explanation. According to him, suicide incidence is higher in spring and summer because social and occupational activities—that mostly takes place at daytime—increases in spring and summer as the days grow longer (20). Indeed, suicide incidence is higher at daytime (13–17), and peaks in suicide incidence shift throughout the year in the same way as the change in timing of sunrise and sunset does (13, 16). For example, the morning peak in suicide incidence happens 3 h earlier in summer than in winter whilst the evening peak in summer happens 3 h later than in winter (16). According to Gabennesch, however, a psychological interpretation might underlie the spring-peak: people have expectations for feeling better at times that might promise a new beginning, such as spring, weekends, or holidays. If, however, these expectations for feeling better promise more than they deliver, it can have a negative effect on subjective well-being, which might become even worse than it previously was. This might result in an increased risk of suicide after the promising event (60).

Currently, seasonality is a well-studied phenomenon in suicide research. Multiple risk factors of suicide were found to be related to seasonal patterns in suicide incidence, however, evidence remains partly inconsistent. Suicide method, occupation, geographic location, allergens, allergy related asthma, rhinitis, and atopic dermatitis are risk factors of suicide that were found to be related to seasonality in suicide (61). For example, with regards to suicide method, suicide rates by violent methods were found to peak in spring and early summer months, and a trough was found in the winter months, which is the same seasonal pattern as suicide incidence (62). More research is needed to the (potential) association of mental disorders, bioclimatic factors (such as sunshine, temperature and rainfall), viruses, pollutants, and month of birth in seasonality of suicide, to better understand the underlying mechanisms of this phenomenon (61).

The third objective is to examine if the incidence of suicide at Christmas differed from other days in December. Our results indicated that nationally, suicide incidence was nearly two times lower at Christmas compared to other days in December. This is in line with international research (11, 18, 19, 38–43). It has been suggested in earlier research that people experience lowered emotional well-being and life satisfaction before Christmas, and alcohol use and psychopathology are increased at Christmas (11, 63), which are all risk factors for suicide; however, this is not confirmed in our study. It is striking that, although several risk factors for suicide are increased at Christmas, the overall utilization of psychiatric emergency services and admissions, the number of self-harm presentations, non-fatal suicide attempts, and completed suicides are all decreased (11, 64).

The broken promise theory of Gabennesch, as already discussed in the previous paragraph, might also be an explanation for this finding (60). Christmas is a holiday that can generate feelings of hope for feeling better in individuals, which might have a protective effect on suicide. However, if Christmas promises more than it actually delivers, the suicide risk might shift from before Christmas, to after (60). This study, in line with previous research, showed an increase in suicide incidence on New Year’s Day and January 2, suggesting that suicides might indeed be delayed until after the Christmas-holidays (18, 19, 40–43).

A second explanation can be that clinicians, family, and friends anticipate that Christmas might be a difficult time for some, which leads to a greater awareness and availability. This increased connectedness and social support might have a protective effect as well (11, 37).

Objective four is to explore if any associations differed in relation to gender, age, and province. In this study, multiple associations between suicide incidence and gender, age, and province were found. With regard to gender, suicide among men occurs more than twice as often than in women. It is well documented that men have higher suicide rates than women (2) and the fact that men are more likely to use violent means and are less likely to seek help than women might be an explanation (65). We found no evidence for a differential effect of season across genders in suicide incidence, which is in line with other studies that found seasonal patterns in suicide incidence in both men and women (21, 23, 25). Yet, some studies found seasonality only or greater present in men (16, 22, 27, 46). Furthermore, the results of this study are in line with previous studies that also failed to find a second minor peak in suicide incidence among women (22, 26, 47), but is in contrast with research that did find a “bimodal distribution” among women (24, 29, 46). Regarding Christmas trends, no evidence for a differential effect of Christmas was found in men and women with regard to suicide incidence. Our finding is in contrast with previous research that did find any gender differences in suicide incidence at Christmas, as other studies found that only men showed significant fewer suicides on Christmas day (19) or reported a greater reduction in women (40, 43).

With regard to age, the results pointed out that suicide rates in the Netherlands are highest in the elderly (aged 80 years and above), which was already well documented (2). An explanation for these high rates in this group may be that older people are increasingly at risk for psychical disabilities, severe pain, depression, loneliness, and social isolation; which are all risk factors for suicide (66, 67). An earlier study in the Netherlands indicated that the median peak in suicide rates among age groups has shifted since 2007 from younger adults to older adults (68). Several factors are known to have a protective effect on suicidal behavior in older people, such as religiosity, life satisfaction, and marriage. Protective factors differ for the old versus the very old population (aged 80 years and above), as it has been found that marriage had no longer a protective effect in people older than 80 years (66). As the Dutch population of 80 years and older is growing since 1995 from 3.1% of the total population to 4.3% in 2015 (69), this might play a role in the rise of suicide rates in the elderly. Furthermore, we found no evidence for a differential effect of season across age groups with regard to suicide incidence. This finding is in line with a previous study from South Africa that also found seasonal variation of suicide in all age groups (23). However, this result is conflicting with multiple other studies that did find associations of age on seasonality in suicide (61).

Regarding provinces, the highest suicide rates were found in Groningen. Groningen was in 2015 the province with the highest unemployment rates (8.5%), which was even 1.6% higher than the national mean (6.9%) (70). Unemployment is a well-known risk factor for suicide (2) and therefore might explain the high suicide rates in this province. However, Zuid-Holland (7.8%) and Flevoland (7.7%) rate second and third, respectively, in 2015 in terms of unemployment rates, but showed the lowest and second-lowest suicide rates (70). We did not find evidence for a differential effect of season in suicide incidence between provinces. These findings were against expectations, since previous research did report differential effects (20, 25, 31, 46). However, previous research mainly focused on urban versus rural areas, whereas this study examined provinces without a distinction in level of urbanization. Therefore, it can be concluded that the seasonal effects on suicide incidence show the same effects in all provinces in the Netherlands. An explanation of this finding might be that the provinces in the Netherlands have too little climatic variation to show differences in seasonal patterns in suicide incidence. Furthermore, no evidence for a differential Christmas effect on suicide incidence between provinces was found. This was against expectation, because Christmas is a Christian holiday and provinces in the Netherlands differ with regards to the size of the Christian-community. An explanation might be that Christmas is also a popular holiday in the non-religious population. As a result, religious trends, such as secularization and the rise of immigrant religions, might have less influence on the extent to which Christmas is celebrated. Therefore, Christmas might be celebrated throughout the country in about the same degree.

### Strengths and Limitations

A strength of this study was the large data set covering all suicides that happened in Dutch residents in 1995–2015, including the demographics gender, age, and province. Since there were no exclusion criteria and drop out was not applicable, this study had no missing data, which is a strength. A limitation of this study is that no daily data were available for age groups; these data were not available to prevent loss of anonymity.

### Implications for Suicide Prevention

Several suicide prevention strategies exist; in creating awareness and education for the general public and professionals, screening tools for at risk individuals, treatment of psychiatric disorders, restricted access to lethal means and responsible media reporting of suicide (1). This study contributes to the first strategy: creating knowledge and awareness about high-risk time frames. The high-risk time frames identified take place in the spring-season and in the beginning of the new year. Although we found a decline in December, and specifically at Christmas, this does not mean that one should be less aware for signals at low-incidence periods, such as Christmas. Suicides do happen at Christmas. Therefore, there must always be awareness for signals. However, we would recommend planning (mental) health care services to be available especially at high-risk moments, which is in spring and in January.

### Implications for Scientific Research

This study has contributed to scientific research by updating and deepening knowledge on time trends in suicide incidence. It also makes corrections possible for naturally occurring time trends in suicide incidence over the year, which is useful when examining the effect of a suicide prevention intervention. In this study, evidence is found that time trends (still) exist in the Netherlands. More research is needed to better understand the underlying mechanisms of time trends in suicide incidence. For example, more research is needed to examine religiousness, urbanization, and method of suicide in relation to time trends. In addition, research is needed to better understand why the effect of Christmas is greater in some provinces and which combination of factors may be more or less favorable in relation to suicide rates. Moreover, we would recommend to examine the effectiveness of certain suicide prevention strategies at these high-risk moments. Multilevel suicide prevention intervention in particular seems to be promising in suicide prevention (71); however, further study on their effectiveness at these specific high-risk moments is needed.

## Author Contributions

EH, IE, MB, JdJ, CN, and CF-C contributed to the conception and design of this paper. EH and MB undertook the statistical analysis. EH wrote the first draft of the paper, and IE, MB, JdJ, CN, and CF-C contributed in the process of drafting and revising. CF-C supervised the procedure and the paper. All authors gave their agreement and approval for all aspects of the final version of the paper.

## Conflict of Interest Statement

The authors declare that the research was conducted in the absence of any commercial or financial relationships that could be construed as a potential conflict of interest.

## Footnotes

^1^Example how we calculated the mean population for 2015: the population at January 1, 2015, and the population at January 1, 2016, were summed and then divided by two.

## References

[1] Mann JJ Apter A Bertolote J Beautrais A Currier D Haas A Suicide prevention strategies: a systematic review. JAMA (2005) 294(16):2064–74. 10.1001/jama.294.16.2064 16249421

[2] WHO. Preventing Suicide: A Global Imperative. Geneva: World Health Organization (2014).

[3] WHO. Age-Standardized Suicide Rates (per 100 000 Population). (2015). Available from: http://www.who.int/gho/mental_health/suicide_rates/en/

[4] Blum RW Nelson-Mmari K. The health of young people in a global context. J Adolesc Health (2004) 35(5):402–18. 10.1016/S1054-139X(03)00537-8 15488435

[5] Wasserman D Cheng Q Jiang G-X. Global suicide rates among young people aged 15–19. World Psychiatry (2005) 4(2):114–20. 16633527 PMC1414751

[6] Gvion Y Apter A Suicide and suicidal behavior. Public Health Rev (2012) 34(2):9 10.1007/BF03391677

[7] CBS. Overledenen; belangrijke doodsoorzaken (korte lijst), regio. (2016). Available from: http://statline.cbs.nl/Statweb/publication/?DM=SLNL&PA=80202NED&D1=88&D2=0&D3=0&D4=0&D5=11-19&HDR=T,G2,G1,G4&STB=G3&VW=T

[8] Theorell T Leymann H Jodko M Konarski K Norbeck HE ’Person under train’ incidents from the subway driver’s point of view – a prospective 1-year follow-up study: the design, and medical and psychiatric data. Soc Sci Med (1994) 38(3):471–5. 10.1016/0277-9536(94)90449-9 8153753

[9] Tranah T Farmer RD. Psychological reactions of drivers to railway suicide. Soc Sci Med (1994) 38(3):459–69. 10.1016/0277-9536(94)90448-0 8153752

[10] Ajdacic-Gross V Lauber C Baumgartner M Malti T Rössler W In-patient suicide – a 13-year assessment. Acta Psychiatr Scand (2009) 120(1):71–5. 10.1111/j.1600-0447.2009.01380.x 19291075

[11] Sansone RA Sansone LA. The Christmas effect on psychopathology. Innov Clin Neurosci (2011) 8(12):10. 22247812 PMC3257984

[12] Christodoulou C Douzenis A Papadopoulos FC Papadopoulou A Bouras G Gournellis R Suicide and seasonality. Acta Psychiatr Scand (2012) 125(2):127–46. 10.1111/j.1600-0447.2011.01750.x 21838741

[13] Van Houwelingen CA Beersma DG. Seasonal changes in 24-h patterns of suicide rates: a study on train suicides in the Netherlands. J Affect Disord (2001) 66(2):215–23. 10.1016/S0165-0327(00)00308-6 11578675

[14] Preti A Miotto P. Diurnal variations in suicide by age and gender in Italy. J Affect Disord (2001) 65(3):253–61. 10.1016/S0165-0327(00)00232-9 11511405

[15] Lukaschek K Baumert J Erazo N Ladwig K-H Stable time patterns of railway suicides in Germany: comparative analysis of 7,187 cases across two observation periods (1995–1998; 2005–2008). BMC Public Health (2014) 14(1):124 10.1186/1471-2458-14-124 24498876 PMC3933256

[16] Erazo N Baumert J Ladwig K-H. Sex-specific time patterns of suicidal acts on the German railway system. An analysis of 4003 cases. J Affect Disord (2004) 83(1):1–9. 10.1016/j.jad.2004.04.012 15546640

[17] Rådbo H Svedung I Andersson R. Suicides and other fatalities from train-person collisions on Swedish railroads: a descriptive epidemiologic analysis as a basis for systems-oriented prevention. J Safety Res (2005) 36(5):423–8. 10.1016/j.jsr.2005.08.003 16303140

[18] Beauchamp GA Ho ML Yin S. Variation in suicide occurrence by day and during major American holidays. J Emerg Med (2014) 46(6):776–81. 10.1016/j.jemermed.2013.09.023 24462023

[19] Zonda T Bozsonyi K Veres E Lester D Frank M The impact of holidays on suicide in Hungary. Omega (Westport) (2009) 58(2):153–62. 10.2190/OM.58.2.e 19227004

[20] Durkheim E In: Spaulding JA Simpson G, editors. Suicide: A Study in Sociology. Glencoe, IL: Free Press (1897). 1951 p.

[21] Postolache TT Mortensen PB Tonelli LH Jiao X Frangakis C Soriano JJ Seasonal spring peaks of suicide in victims with and without prior history of hospitalization for mood disorders. J Affect Disord (2010) 121(1):88–93. 10.1016/j.jad.2009.05.015 19535151 PMC2837087

[22] Zonda T Bozsonyi K Veres E Seasonal fluctuation of suicide in Hungary between 1970–2000. Arch Suicide Res (2005) 9(1):77–85. 10.1080/13811110590512967 16040582

[23] Flisher AJ Parry CD Bradshaw D Juritz JM Seasonal variation of suicide in South Africa. Psychiatry Res (1997) 66(1):13–22. 10.1016/S0165-1781(96)02974-5 9061800

[24] Preti A Miotto P Seasonality in suicides: the influence of suicide method, gender and age on suicide distribution in Italy. Psychiatry Res (1998) 81(2):219–31. 10.1016/S0165-1781(98)00099-7 9858038

[25] Sun J Guo X Ma J Zhang J Jia C Xu A Seasonality of suicide in Shandong China, 1991–2009: associations with gender, age, area and methods of suicide. J Affect Disord (2011) 135(1):258–66. 10.1016/j.jad.2011.08.008 21875753

[26] Ho TP Chao A Yip P Seasonal variation in suicides re-examined: no sex difference in Hong Kong and Taiwan. Acta Psychiatr Scand (1997) 95(1):26–31. 10.1111/j.1600-0447.1997.tb00369.x 9051157

[27] Preti A. The influence of seasonal change on suicidal behaviour in Italy. J Affect Disord (1997) 44(2):123–30. 10.1016/S0165-0327(97)00035-9 9241572

[28] Kevan SM Perspectives on season of suicide: a review. Soc Sci Med Med Geogr (1980) 14(4):369–78. 7006089 10.1016/0160-8002(80)90005-2

[29] Rihmer Z Rutz W Pihlgren H Pestality P. Decreasing tendency of seasonality in suicide may indicate lowering rate of depressive suicides in the population. Psychiatry Res (1998) 81(2):233–40. 10.1016/S0165-1781(98)00106-1 9858039

[30] Preti A Miotto P Coppi MD. Season and suicide: recent findings from Italy. Crisis (2000) 21(2):59. 10.1027//0227-5910.21.2.59 11019481

[31] Ajdacic-Gross V Bopp M Sansossio R Lauber C Gostynski M Eich D Diversity and change in suicide seasonality over 125 years. J Epidemiol Community Health (2005) 59(11):967–72. 10.1136/jech.2004.030981 16234425 PMC1732944

[32] Mackenbach J Kunst A Looman C Seasonal variation in mortality in the Netherlands. J Epidemiol Community Health (1992) 46(3):261–5. 10.1136/jech.46.3.261 1645083 PMC1059564

[33] Chew KS McCleary R. The spring peak in suicides: a cross-national analysis. Soc Sci Med (1995) 40(2):223–30. 10.1016/0277-9536(94)E0070-9 7899934

[34] APP. Nearly Half of News Stories Still Making the False Holiday-Suicide Connection. (2016).

[35] Melrose S. Seasonal affective disorder: an overview of assessment and treatment approaches. Depress Res Treat (2015) 2015:178564. 10.1155/2015/178564 26688752 PMC4673349

[36] Lam RW Levitan RD. Pathophysiology of seasonal affective disorder: a review. J Psychiatry Neurosci (2000) 25(5):469–80. 11109298 PMC1408021

[37] Vreeman RC Carroll AE Festive medical myths. BMJ (2008) 337:a2769 10.1136/bmj.a2769 19091758

[38] Carley S Hamilton M Suicide at Christmas. Emerg Med J (2004) 21(6):716–7. 10.1136/emj.2004.019703 15496706 PMC1726490

[39] Ajdacic-Gross V Wang J Bopp M Eich D Rössler W Gutzwiller F. Are seasonalities in suicide dependent on suicide methods? A reappraisal. Soc Sci Med (2003) 57(7):1173–81. 10.1016/S0277-9536(02)00493-8 12899902

[40] Jessen G Jensen BF. Postponed suicide death? Suicides around birthdays and major public holidays. Suicide Life Threat Behav (1999) 29(3):272–83. 10531639

[41] Bridges FS. Rates of homicide and suicide on major national holidays. Psychol Rep (2004) 94(2):723–4. 10.2466/pr0.94.2.723-724 15154207

[42] Plöderl M Fartacek C Kunrath S Pichler E-M Fartacek R Datz C Nothing like Christmas – suicides during Christmas and other holidays in Austria. Eur J Public Health (2014) 25(3):410–3. 10.1093/eurpub/cku169 25245117

[43] Cavanagh B Ibrahim S Roscoe A Bickley H While D Windfuhr K The timing of general population and patient suicide in England, 1997–2012. J Affect Disord (2016) 197:175–81. 10.1016/j.jad.2016.02.055 26994435

[44] Demirci S Dogan KH Koc S. Evaluation of forensic deaths during the month of Ramadan in Konya, Turkey, between 2000 and 2009. Am J Forensic Med Pathol (2013) 34(3):267–70. 10.1097/PAF.0b013e3182a0a430 23883868

[45] Knippenberg H Secularisation and the rise of immigrant religions: the case of the Netherlands. Acta Univ Carol Geogr (2010) 44(1–2):63–82.

[46] Micciolo R Williams P Zimmermann-Tansella C Tansella M Geographical and urban – rural variation in the seasonality of suicide: some further evidence. J Affect Disord (1991) 21(1):39–43. 10.1016/0165-0327(91)90017-M 1827475

[47] Yip PS Chao A Ho T. A re-examination of seasonal variation in suicides in Australia and New Zealand. J Affect Disord (1998) 47(1):141–50. 10.1016/S0165-0327(97)00135-3 9476754

[48] CBS. Niet-natuurlijk overlijden. Available from: https://www.cbs.nl/nl-nl/onze-diensten/methoden/onderzoeksomschrijvingen/korte-onderzoeksbeschrijvingen/niet-natuurlijk-overlijden

[49] Värnik P Sisask M Värnik A Laido Z Meise U Ibelshäuser A Suicide registration in eight European countries: a qualitative analysis of procedures and practices. Forensic Sci Int (2010) 202(1):86–92. 10.1016/j.forsciint.2010.04.032 20483553

[50] World Health Organization. International Statistical Classification of Diseases and Related Health Problems. 10th Revision (ICD-10). Geneva: World Health Organization (2011).

[51] Linsley KR Schapira K Kelly T Open verdict v. suicide – importance to research. Br J Psychiatry (2001) 178(5):465–8. 10.1192/bjp.178.5.465 11331564

[52] Birt C Bille-Brahe U Cabecadas M Chishti P Corcoran P Elgie R Suicide mortality in the European Union. Eur J Public Health (2003) 13(2):108–14. 10.1093/eurpub/13.2.108 12803408

[53] Bertolote JM Fleischmann A A global perspective in the epidemiology of suicide. Suicidologi (2015) 7(2):6–8. 10.5617/suicidologi.2330

[54] Breiding MJ Wiersema B. Variability of undetermined manner of death classification in the US. Inj Prev (2006) 12(Suppl 2):ii49–54. 10.1136/ip.2006.012591 17170172 PMC2563487

[55] Von Elm E Altman DG Egger M Pocock SJ Gøtzsche PC Vandenbroucke JP The strengthening the reporting of observational studies in epidemiology (STROBE) statement: guidelines for reporting observational studies. Int J Surg (2014) 12(12):1495–9. 10.1016/j.ijsu.2014.07.013 25046131

[56] Martin-Carrasco M Evans-Lacko S Dom G Christodoulou N Samochowiec J Gonzalez-Fraile E EPA guidance on mental health and economic crises in Europe. Eur Arch Psychiatry Clin Neurosci (2016) 266(2):89–124. 10.1007/s00406-016-0681-x 26874960

[57] Chang S-S Stuckler D Yip P Gunnell D. Impact of 2008 global economic crisis on suicide: time trend study in 54 countries. BMJ (2013) 347:f5239. 10.1136/bmj.f5239 24046155 PMC3776046

[58] Stuckler D Basu S Suhrcke M Coutts A McKee M. The public health effect of economic crises and alternative policy responses in Europe: an empirical analysis. Lancet (2009) 374(9686):315–23. 10.1016/S0140-6736(09)61124-7 19589588

[59] Economou M Angelopoulos E Peppou LE Souliotis K Stefanis C Suicidal ideation and suicide attempts in Greece during the economic crisis: an update. World Psychiatry (2016) 15(1):83–4. 10.1002/wps.20296 26833617 PMC4780297

[60] Gabennesch H When promises fail: a theory of temporal fluctuations in suicide. Soc Forces (1988) 67:129–45. 10.1093/sf/67.1.129

[61] Woo J-M Okusaga O Postolache TT. Seasonality of suicidal behavior. Int J Environ Res Public Health (2012) 9(2):531–47. 10.3390/ijerph9020531 22470308 PMC3315262

[62] Hakko H Räsänen P Tiihonen J. Seasonal variation in suicide occurrence in Finland. Acta Psychiatr Scand (1998) 98(2):92–7. 10.1111/j.1600-0447.1998.tb10048.x 9718233

[63] Mutz M Christmas and subjective well-being: a research note. Appl Res Qual Life (2016) 11(4):1341–56. 10.1007/s11482-015-9441-8

[64] Griffin E Dillon CB O’Regan G Corcoran P Perry IJ Arensman E. The paradox of public holidays: hospital-treated self-harm and associated factors. J Affect Disord (2017) 218:30–4. 10.1016/j.jad.2017.04.058 28456074

[65] Hawton K Sex and suicide. Gender differences in suicidal behavior. Br J Psychiatry (2000) 177(6):484–5. 10.1192/bjp.177.6.484 11102320

[66] O’Connell H Chin A-V Cunningham C Lawlor BA Recent developments: suicide in older people. BMJ (2004) 329(7471):895 10.1136/bmj.329.7471.895 15485967 PMC523116

[67] Juurlink DN Herrmann N Szalai JP Kopp A Redelmeier DA. Medical illness and the risk of suicide in the elderly. Arch Intern Med (2004) 164(11):1179–84. 10.1001/archinte.164.11.1179 15197042

[68] de Beurs DP Hooiveld M Kerkhof AJ Korevaar JC Donker GA Trends in suicidal behaviour in Dutch general practice 1983–2013: a retrospective observational study. BMJ Open (2016) 6(5):e010868 10.1136/bmjopen-2015-010868 PMC4874133 27165647

[69] CBS. Bevolking; Kerncijfers. (2017). Available from: http://statline.cbs.nl/Statweb/publication/?DM=SLNL&PA=37296ned&D1=14-18&D2=45-65&HDR=G1&STB=T&VW=T

[70] CBS. Arbeidsdeelname; Provincie. (2015). Available from: http://statline.cbs.nl/Statweb/publication/?DM=SLNL&PA=83523ned&D1=12&D2=0,5-16&D3=64,l&HDR=G2,T&STB=G1&VW=T

[71] Van der Feltz-Cornelis CM Sarchiapone M Postuvan V Volker D Roskar S Grum AT Best practice elements of multilevel suicide prevention strategies. Crisis (2011) 23(6):319–33. 10.1027/0227-5910/a000109 PMC3306243 21945840

